# Ba_2_Sb_4_GeS_10_

**DOI:** 10.1107/S1600536813007988

**Published:** 2013-04-05

**Authors:** Lei Geng

**Affiliations:** a Department of Physics and Electronic Information, Huaibei Normal University, Huaibei, Anhui 235000, People’s Republic of China

## Abstract

The title quaternary compound, dibarium tetra­anti­mony germanium deca­sulfide, Ba_2_Sb_4_GeS_10_, crystallizes in a novel three-dimensional _∞_^3^[Sb_4_GeS_10_]^4−^ network structure, which is composed of triangular pyramidal SbS_3_ (site symmetry *m*..), distorted SbS_5_ (*m*..) polyhedra and regular GeS_4_ (-4..) tetra­hedra. The SbS_3_ and SbS_5_ units are connected with each other through corner- and edge-sharing, forming a Sb_4_S_10_ layer in the *ab* plane. The GeS_4_ tetra­hedra further bridge two neighbouring Sb_4_S_10_ layers, forming a three-dimensional _∞_^3^[Sb_4_GeS_10_]^4−^ network. The Ba^2+^ cation (..2) is located between two Sb_4_S_10_ layers and is coordinated by ten S atoms with Ba—S bond lengths in the range 3.2505 (9)–3.4121 (2) Å.

## Related literature

The stereochemically active 5*s* ^2^ lone-pair electrons possess a large electric dipole moment and can influence structures that contain Sb^3+^, see: Choi & Kanatzidis (2000 ▶); Babo & Albrecht-Schmitt (2012 ▶). SbS_3_, SbS_4_ or SbS_5_ units in a crystal structure are prone to form Sb—S chains through corner- or edge-sharing, see: Dorrscheidt & Schäfer (1981 ▶); Cordier *et al.* (1984 ▶). GeS_4_ tetra­hedra can be utilized as the second structural unit and introduced into crystal structures to connect Sb—S chains into a two-dimensional layer or three-dimensional framework structure (Feng *et al.*, 2008 ▶). For crystal structures and optical properties, see: Deng *et al.* (2005 ▶); Kim *et al.* (2008 ▶); Ribes *et al.* (1973 ▶); Teske (1979 ▶); Lekse *et al.* (2009 ▶).

## Experimental

### 

#### Crystal data

Ba_2_Sb_4_GeS_10_
*M* *_r_* = 1154.87
Tetragonal, 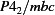

*a* = 11.3119 (4) Å
*c* = 13.6384 (9) Å
*V* = 1745.16 (14) Å^3^
*Z* = 4
Mo *K*α radiation
μ = 13.40 mm^−1^
*T* = 293 K
0.22 × 0.07 × 0.07 mm

#### Data collection

Rigaku SCXMini CCD diffractometer
Absorption correction: multi-scan (*CrystalClear*; Rigaku, 2007 ▶) *T*_min_ = 0.530, *T*_max_ = 1.000
12411 measured reflections
1046 independent reflections
1032 reflections with *I* > 2σ(*I*)
*R*_int_ = 0.030

#### Refinement

*R*[*F* ^2^ > 2σ(*F* ^2^)] = 0.018
*wR*(*F* ^2^) = 0.042
*S* = 1.15
1046 reflections
45 parameters
Δρ_max_ = 0.66 e Å^−3^
Δρ_min_ = −0.74 e Å^−3^

### 

Data collection: *CrystalClear* (Rigaku, 2007 ▶); cell refinement: *CrystalClear*; data reduction: *CrystalClear*; program(s) used to solve structure: *SHELXS97* (Sheldrick, 2008 ▶); program(s) used to refine structure: *SHELXL97* (Sheldrick, 2008 ▶); molecular graphics: *DIAMOND* (Brandenburg, 2005 ▶); software used to prepare material for publication: *publCIF* (Westrip, 2010 ▶).

## Supplementary Material

Crystal structure: contains datablock(s) I, global. DOI: 10.1107/S1600536813007988/jj2163sup1.cif

Structure factors: contains datablock(s) I. DOI: 10.1107/S1600536813007988/jj2163Isup2.hkl

Additional supplementary materials:  crystallographic information; 3D view; checkCIF report

## Figures and Tables

**Fig. 1.**
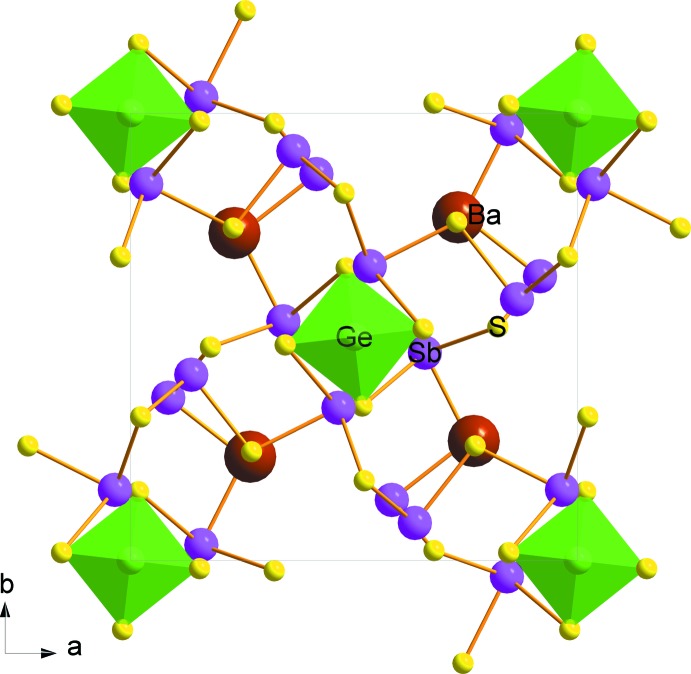
Crystal structure of Ba2Sb4GeS10 in GeS4 polyhedral representation viewed along [001]. Green tetrahedra represent the GeS4 unit with yellow sulfur atoms at the corners of each tetrahedron.

**Fig. 2.**
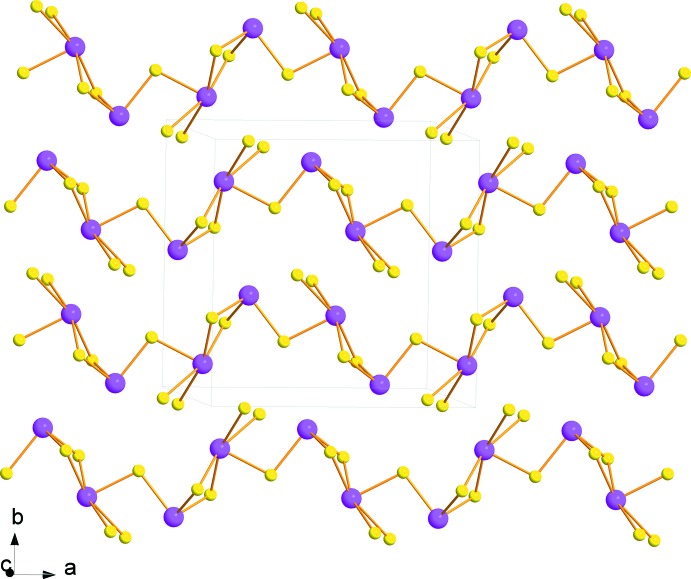
SbS3 and SbS5 polyhedra are connected with each other through corner- and edge-sharing conformations to form the Sb4S10 zigzag chains, which are further arranged side by side into the Sb4S10 layer in the ab-plane.

**Fig. 3.**
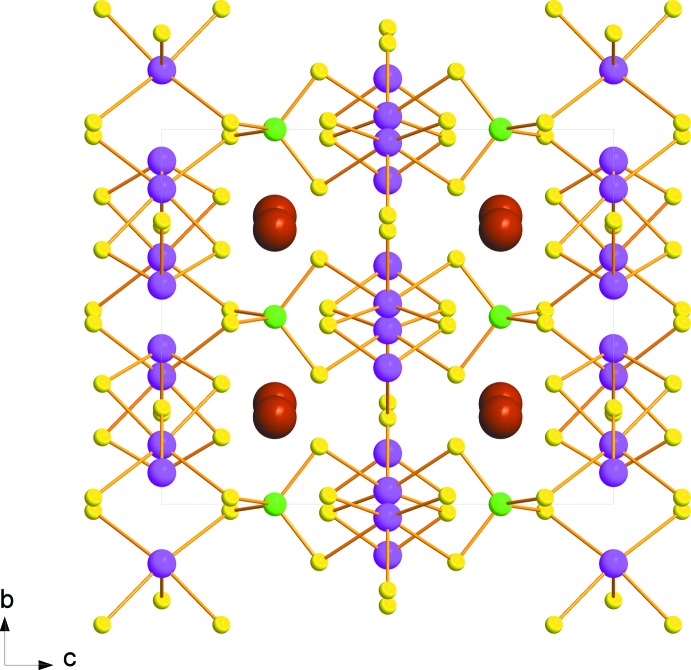
The novel three-dimensional ∞3[Sb4GeS10]-4 network viewed along [100] direction. GeS4 tetrahedra act as bridging units for two neighbouring Sb4S10 layers.

## supplementary crystallographic information

### Comment

Sb-based chalcogenides have attracted a lot of attention in recent years due to their rich structures and interesting physical properties, such as nonlinear optics and ion-exchange properties. The stereochemically active 5 s^2^ lone-pair electrons possess a large electric dipole moment and can influence structures that contain Sb^3+^ (Choi *et al.* 2000; Babo *et al.* 2012). SbS_3_, SbS_4_ or SbS_5_ units in a crystal structure are prone to form one-dimensional Sb—S chains through a corner- or edge-sharing manner (Dorrscheidt *et al.* 1981; Cordier *et al.* 1984). GeS_4_ tetrahedron can be utilized as the second structural unit and introduced into crystal structure to connect Sb—S chains into a two-dimensional layer or three-dimensional framework structure (Feng *et al.* 2008). In this paper, a new title quaternary sulfide in the Sb—Ge—S system is dexcribed. Ba_2_Sb_4_GeS_10_ represents the first example of a structure synthesized and structurally characterized by single-crystal X-ray diffraction in the quaternary Ba—Sb—Ge—S system (Fig. 1). It crystallizes with one Ge atom on 4*b* and three S atoms on 8*h*, 16*i* and 16*i*, respectively, in terms of the Wyckoff notation. There are two types of coordination polyhedron of SbS groups, *i.e.* triangle-pyramidal SbS_3_ and distorted SbS_5_ polyhedra. SbS_3_ and SbS_5_ polyhedra are connected with each other through corner- and edge-sharing conformations to form Sb_4_S_10_ zigzag chains, which are further arranged side by side into the Sb_4_S_10_ layer in the *ab*-plane (Fig. 2). The GeS_4_ tetrahedra further bridge two neighbouring Sb_4_S_10_ layers, forming athree-dimensional ∞^3^[Sb_4_GeS_10_]^-4^ network (Fig. 3). The Ba atom is located between two Sb_4_S_10_ layers and coordinates with ten S atoms with Ba—S bonding lengths in the range of 3.2505 (9)–3.4121 (2) Å, which are typical values for sulfides (Dorrscheidt *et al.* 1981; Cordier *et al.* 1984).

### Experimental

The title compound, Ba_2_Sb_4_GeS_10_, was synthesized through a high temperature solid-state reaction in an evacuated and sealed silica tube. A mixture of BaS (0.5 mmol, 0.0847 g), Sb (1 mmol, 0.1218 g), Ge (0.25 mmol, 0.0182 g),S (2 mmol, 0.0641 g) was loaded in a silica ampoule, sealed under 10 ^-2^ Pa, heated gradually to 1173 K (holding for 10 h) in 60 h, and then cooled to room temperature in 300 h. Rod-shaped crystals of Ba_2_Sb_4_GeS_10_ with a dark red color were obtained. The crystals were stable under air and moisture conditions.

### Refinement

All atoms in Ba_2_Sb_4_GeS_10_ crystal structure were refined anisotropically without disorder. The highest residual peak (0.66 e×Å^-3^) in the difference electron density map was located at (0.1992, 0.3008, 1/4), 1.09 Å from atom Ba1. The deepest hole (- 0.73 e×Å^-3^) was located at (0.1670, 0.7496, 0.2499), 0.77 Å from atom Ba1.

### Crystal data

**Table:**

Ba_2_Sb_4_GeS_10_	*D*_x_ = 4.395 Mg m^−^^3^
*M**_r_* = 1154.87	Mo *K*α radiation, λ = 0.71073 Å
Tetragonal, *P*4_2_/*m**b**c*	Cell parameters from 1877 reflections
Hall symbol: -P 4ac 2ab	θ = 2.3–27.5°
*a* = 11.3119 (4) Å	µ = 13.40 mm^−^^1^
*c* = 13.6384 (9) Å	*T* = 293 K
*V* = 1745.16 (14) Å^3^	Rod, dark-red
*Z* = 4	0.22 × 0.07 × 0.07 mm
*F*(000) = 2032	

### Data collection

**Table:**

Rigaku SCXMini CCD diffractometer	1046 independent reflections
Radiation source: fine-focus sealed tube	1032 reflections with *I* > 2σ(*I*)
Graphite monochromator	*R*_int_ = 0.030
CCD_Profile_fitting scans	θ_max_ = 27.5°, θ_min_ = 2.6°
Absorption correction: multi-scan (*CrystalClear*; Rigaku, 2007)	*h* = −14→14
*T*_min_ = 0.530, *T*_max_ = 1.000	*k* = −14→11
12411 measured reflections	*l* = −16→17

### Refinement

**Table:**

Refinement on *F*^2^	Primary atom site location: structure-invariant direct methods
Least-squares matrix: full	Secondary atom site location: difference Fourier map
*R*[*F*^2^ > 2σ(*F*^2^)] = 0.018	*w* = 1/[σ^2^(*F*_o_^2^) + (0.0214*P*)^2^ + 3.2912*P*] where *P* = (*F*_o_^2^ + 2*F*_c_^2^)/3
*wR*(*F*^2^) = 0.042	(Δ/σ)_max_ < 0.001
*S* = 1.15	Δρ_max_ = 0.66 e Å^−^^3^
1046 reflections	Δρ_min_ = −0.74 e Å^−^^3^
45 parameters	Extinction correction: *SHELXL97* (Sheldrick, 2008), Fc^*^=kFc[1+0.001xFc^2^λ^3^/sin(2θ)]^-1/4^
0 restraints	Extinction coefficient: 0.00225 (8)

### Special details

**Table:**

Geometry. All e.s.d.'s (except the e.s.d. in the dihedral angle between two l.s. planes) are estimated using the full covariance matrix. The cell e.s.d.'s are taken into account individually in the estimation of e.s.d.'s in distances, angles and torsion angles; correlations between e.s.d.'s in cell parameters are only used when they are defined by crystal symmetry. An approximate (isotropic) treatment of cell e.s.d.'s is used for estimating e.s.d.'s involving l.s. planes.
Refinement. Refinement of *F*^2^ against ALL reflections. The weighted *R*-factor *wR* and goodness of fit *S* are based on *F*^2^, conventional *R*-factors *R* are based on *F*, with *F* set to zero for negative *F*^2^. The threshold expression of *F*^2^ > σ(*F*^2^) is used only for calculating *R*-factors(gt) *etc*. and is not relevant to the choice of reflections for refinement. *R*-factors based on *F*^2^ are statistically about twice as large as those based on *F*, and *R*- factors based on ALL data will be even larger.

### Fractional atomic coordinates and isotropic or equivalent isotropic displacement parameters (Å^2^)

**Table:**

	*x*	*y*	*z*	*U*_iso_*/*U*_eq_	
Ba1	0.232424 (19)	0.732424 (19)	0.2500	0.01704 (10)	
Sb1	0.13578 (3)	0.41567 (3)	0.0000	0.01543 (10)	
Sb2	0.46488 (3)	0.34169 (3)	0.0000	0.01796 (10)	
Ge1	0.0000	0.0000	0.2500	0.01175 (15)	
S1	0.27060 (10)	0.24355 (10)	0.0000	0.0136 (2)	
S2	0.01781 (8)	0.15428 (7)	0.34843 (6)	0.01631 (18)	
S3	0.02261 (7)	0.32137 (8)	0.13186 (6)	0.01777 (19)	

### Atomic displacement parameters (Å^2^)

**Table:**

	*U*^11^	*U*^22^	*U*^33^	*U*^12^	*U*^13^	*U*^23^
Ba1	0.01715 (12)	0.01715 (12)	0.01683 (15)	0.00214 (11)	−0.00097 (7)	0.00097 (7)
Sb1	0.01518 (17)	0.01341 (17)	0.01771 (17)	0.00143 (11)	0.000	0.000
Sb2	0.01151 (17)	0.02030 (18)	0.02206 (18)	−0.00225 (12)	0.000	0.000
Ge1	0.0126 (2)	0.0126 (2)	0.0101 (3)	0.000	0.000	0.000
S1	0.0101 (5)	0.0121 (5)	0.0184 (6)	−0.0006 (4)	0.000	0.000
S2	0.0201 (4)	0.0142 (4)	0.0146 (4)	−0.0020 (3)	−0.0009 (3)	−0.0026 (3)
S3	0.0131 (4)	0.0254 (4)	0.0147 (4)	−0.0025 (3)	0.0024 (3)	−0.0028 (3)

### Geometric parameters (Å, º)

**Table:**

Ba1—S2^i^	3.2505 (9)	Sb2—S3^viii^	2.6576 (9)
Ba1—S2^ii^	3.2505 (9)	Sb2—S2^iv^	2.9352 (9)
Ba1—S3^ii^	3.3596 (9)	Sb2—S2^ix^	2.9352 (9)
Ba1—S3^i^	3.3596 (9)	Ge1—S2	2.2110 (8)
Ba1—S3^iii^	3.3599 (8)	Ge1—S2^x^	2.2110 (8)
Ba1—S3^iv^	3.3599 (8)	Ge1—S2^xi^	2.2110 (8)
Ba1—S2^iv^	3.3849 (9)	Ge1—S2^xii^	2.2110 (8)
Ba1—S2^iii^	3.3849 (9)	S1—Ba1^xiii^	3.4121 (2)
Ba1—S1^ii^	3.4121 (2)	S1—Ba1^xiv^	3.4121 (2)
Ba1—S1^v^	3.4121 (2)	S2—Sb2^xv^	2.9352 (9)
Sb1—S3^vi^	2.4517 (9)	S2—Ba1^xiv^	3.2505 (9)
Sb1—S3	2.4517 (9)	S2—Ba1^iii^	3.3849 (9)
Sb1—S1	2.4732 (12)	S3—Sb2^xvi^	2.6576 (9)
Sb2—S1	2.4621 (12)	S3—Ba1^xiv^	3.3596 (9)
Sb2—S3^vii^	2.6576 (9)	S3—Ba1^iii^	3.3599 (8)
			
S2^i^—Ba1—S2^ii^	130.04 (3)	S3^iv^—Ba1—S1^v^	120.60 (3)
S2^i^—Ba1—S3^ii^	76.46 (2)	S2^iv^—Ba1—S1^v^	68.80 (2)
S2^ii^—Ba1—S3^ii^	64.06 (2)	S2^iii^—Ba1—S1^v^	111.96 (2)
S2^i^—Ba1—S3^i^	64.06 (2)	S1^ii^—Ba1—S1^v^	177.82 (4)
S2^ii^—Ba1—S3^i^	76.46 (2)	S3^vi^—Sb1—S3	94.37 (4)
S3^ii^—Ba1—S3^i^	74.69 (3)	S3^vi^—Sb1—S1	88.82 (3)
S2^i^—Ba1—S3^iii^	153.52 (2)	S3—Sb1—S1	88.82 (3)
S2^ii^—Ba1—S3^iii^	74.09 (2)	S1—Sb2—S3^vii^	84.63 (3)
S3^ii^—Ba1—S3^iii^	129.53 (3)	S1—Sb2—S3^viii^	84.63 (3)
S3^i^—Ba1—S3^iii^	122.17 (3)	S3^vii^—Sb2—S3^viii^	85.17 (4)
S2^i^—Ba1—S3^iv^	74.09 (2)	S1—Sb2—S2^iv^	80.46 (3)
S2^ii^—Ba1—S3^iv^	153.52 (2)	S3^vii^—Sb2—S2^iv^	164.84 (3)
S3^ii^—Ba1—S3^iv^	122.17 (3)	S3^viii^—Sb2—S2^iv^	90.69 (3)
S3^i^—Ba1—S3^iv^	129.53 (3)	S1—Sb2—S2^ix^	80.46 (3)
S3^iii^—Ba1—S3^iv^	85.35 (3)	S3^vii^—Sb2—S2^ix^	90.69 (3)
S2^i^—Ba1—S2^iv^	66.88 (3)	S3^viii^—Sb2—S2^ix^	164.84 (3)
S2^ii^—Ba1—S2^iv^	131.76 (3)	S2^iv^—Sb2—S2^ix^	89.54 (3)
S3^ii^—Ba1—S2^iv^	140.05 (2)	S2—Ge1—S2^x^	111.63 (2)
S3^i^—Ba1—S2^iv^	75.40 (2)	S2—Ge1—S2^xi^	105.23 (4)
S3^iii^—Ba1—S2^iv^	89.05 (2)	S2^x^—Ge1—S2^xi^	111.63 (2)
S3^iv^—Ba1—S2^iv^	62.66 (2)	S2—Ge1—S2^xii^	111.63 (2)
S2^i^—Ba1—S2^iii^	131.76 (3)	S2^x^—Ge1—S2^xii^	105.23 (4)
S2^ii^—Ba1—S2^iii^	66.88 (3)	S2^xi^—Ge1—S2^xii^	111.63 (2)
S3^ii^—Ba1—S2^iii^	75.40 (2)	Sb2—S1—Sb1	101.27 (4)
S3^i^—Ba1—S2^iii^	140.05 (2)	Sb2—S1—Ba1^xiii^	91.466 (19)
S3^iii^—Ba1—S2^iii^	62.66 (2)	Sb1—S1—Ba1^xiii^	91.31 (2)
S3^iv^—Ba1—S2^iii^	89.05 (2)	Sb2—S1—Ba1^xiv^	91.466 (19)
S2^iv^—Ba1—S2^iii^	142.24 (3)	Sb1—S1—Ba1^xiv^	91.31 (2)
S2^i^—Ba1—S1^ii^	63.42 (2)	Ba1^xiii^—S1—Ba1^xiv^	175.62 (4)
S2^ii^—Ba1—S1^ii^	115.56 (2)	Ge1—S2—Sb2^xv^	96.58 (3)
S3^ii^—Ba1—S1^ii^	61.18 (2)	Ge1—S2—Ba1^xiv^	92.47 (3)
S3^i^—Ba1—S1^ii^	116.86 (3)	Sb2^xv^—S2—Ba1^xiv^	86.85 (2)
S3^iii^—Ba1—S1^ii^	120.60 (3)	Ge1—S2—Ba1^iii^	88.96 (3)
S3^iv^—Ba1—S1^ii^	61.24 (2)	Sb2^xv^—S2—Ba1^iii^	154.96 (3)
S2^iv^—Ba1—S1^ii^	111.96 (2)	Ba1^xiv^—S2—Ba1^iii^	117.39 (2)
S2^iii^—Ba1—S1^ii^	68.80 (2)	Sb1—S3—Sb2^xvi^	86.21 (3)
S2^i^—Ba1—S1^v^	115.56 (2)	Sb1—S3—Ba1^xiv^	92.95 (3)
S2^ii^—Ba1—S1^v^	63.42 (2)	Sb2^xvi^—S3—Ba1^xiv^	108.63 (3)
S3^ii^—Ba1—S1^v^	116.86 (3)	Sb1—S3—Ba1^iii^	151.51 (4)
S3^i^—Ba1—S1^v^	61.18 (2)	Sb2^xvi^—S3—Ba1^iii^	89.30 (2)
S3^iii^—Ba1—S1^v^	61.24 (2)	Ba1^xiv^—S3—Ba1^iii^	115.09 (3)
			
S3^vii^—Sb2—S1—Sb1	−137.18 (2)	S3—Sb1—S1—Ba1^xiv^	−41.05 (3)
S3^viii^—Sb2—S1—Sb1	137.18 (2)	S2^x^—Ge1—S2—Sb2^xv^	157.74 (2)
S2^iv^—Sb2—S1—Sb1	45.572 (18)	S2^xi^—Ge1—S2—Sb2^xv^	36.474 (13)
S2^ix^—Sb2—S1—Sb1	−45.572 (18)	S2^xii^—Ge1—S2—Sb2^xv^	−84.791 (7)
S3^vii^—Sb2—S1—Ba1^xiii^	−45.55 (3)	S2^x^—Ge1—S2—Ba1^xiv^	−115.15 (4)
S3^viii^—Sb2—S1—Ba1^xiii^	−131.19 (3)	S2^xi^—Ge1—S2—Ba1^xiv^	123.59 (3)
S2^iv^—Sb2—S1—Ba1^xiii^	137.20 (3)	S2^xii^—Ge1—S2—Ba1^xiv^	2.32 (3)
S2^ix^—Sb2—S1—Ba1^xiii^	46.06 (2)	S2^x^—Ge1—S2—Ba1^iii^	2.23 (2)
S3^vii^—Sb2—S1—Ba1^xiv^	131.19 (3)	S2^xi^—Ge1—S2—Ba1^iii^	−119.04 (3)
S3^viii^—Sb2—S1—Ba1^xiv^	45.55 (3)	S2^xii^—Ge1—S2—Ba1^iii^	119.70 (3)
S2^iv^—Sb2—S1—Ba1^xiv^	−46.06 (2)	S3^vi^—Sb1—S3—Sb2^xvi^	22.13 (4)
S2^ix^—Sb2—S1—Ba1^xiv^	−137.20 (3)	S1—Sb1—S3—Sb2^xvi^	−66.60 (3)
S3^vi^—Sb1—S1—Sb2	132.80 (2)	S3^vi^—Sb1—S3—Ba1^xiv^	130.615 (18)
S3—Sb1—S1—Sb2	−132.80 (2)	S1—Sb1—S3—Ba1^xiv^	41.89 (3)
S3^vi^—Sb1—S1—Ba1^xiii^	41.05 (3)	S3^vi^—Sb1—S3—Ba1^iii^	−59.38 (9)
S3—Sb1—S1—Ba1^xiii^	135.44 (3)	S1—Sb1—S3—Ba1^iii^	−148.11 (7)
S3^vi^—Sb1—S1—Ba1^xiv^	−135.44 (3)		

Symmetry codes: (i) −*x*+1/2, *y*+1/2, *z*; (ii) *y*, −*x*+1, −*z*+1/2; (iii) −*x*, −*y*+1, *z*; (iv) −*y*+1/2, −*x*+1/2, −*z*+1/2; (v) −*x*+1/2, *y*+1/2, −*z*; (vi) *x*, *y*, −*z*; (vii) *x*+1/2, −*y*+1/2, −*z*; (viii) *x*+1/2, −*y*+1/2, *z*; (ix) −*y*+1/2, −*x*+1/2, *z*−1/2; (x) −*y*, *x*, −*z*+1/2; (xi) −*x*, −*y*, *z*; (xii) *y*, −*x*, −*z*+1/2; (xiii) −*y*+1, *x*, *z*−1/2; (xiv) −*y*+1, *x*, −*z*+1/2; (xv) −*y*+1/2, −*x*+1/2, *z*+1/2; (xvi) *x*−1/2, −*y*+1/2, *z*.
